# Adolescent Total and Mental Health–Related Emergency Department Visits During the COVID-19 Pandemic

**DOI:** 10.1001/jamanetworkopen.2023.36463

**Published:** 2023-10-05

**Authors:** Sofia B. Villas-Boas, Scott Kaplan, Justin S. White, Renee Y. Hsia

**Affiliations:** 1 Department of Agricultural and Resource Economics, College of Natural Resources, University of California, Berkeley; 2 Department of Economics, US Naval Academy, Annapolis, Maryland; 3 Department of Health Law, Policy & Management, Boston University School of Public Health, Boston, Massachusetts; 4 Department of Epidemiology & Biostatistics, University of California, San Francisco; 5 Department of Emergency Medicine, University of California, San Francisco; 6 Philip R. Lee Institute for Health Policy Studies, University of California, San Francisco

## Abstract

**Importance:**

Previous research has assessed changes in pediatric and adolescent health care utilization during the COVID-19 pandemic; however, less is known regarding how the pandemic affected adolescents’ use of emergency care, specifically for mental health (MH).

**Objective:**

To determine how adolescents (ages 12-17 years), compared with other age groups, sought help in emergency departments (EDs) in general and for MH conditions during the COVID-19 pandemic.

**Design and Setting:**

In this cross-sectional study, National Syndromic Surveillance Program data and the Centers for Disease Control and Prevention Mental Health, version 1, query were used to track patterns in weekly adolescent ED visits by region across the 10 US Department of Health and Human Services regions from January 2019 through December 2021. Data analysis was performed in April and May 2023.

**Main Outcomes and Measures:**

Total ED visits, MH-related ED visits, and the proportion of total ED visits that were MH related by week-region.

**Results:**

Both weekly regional ED visits and MH-related ED visits dropped after the onset of the pandemic. Because total ED visits dropped more than MH-related ED visits, the proportion of MH-related ED visits increased for the adolescent group. Total ED visits only returned to prepandemic weekly levels (2019: mean [range], 7358 [715-25 908] visits) in the middle of 2021 (overall in 2021: mean [range], 6210 [623-25 777] visits). Mental health–related visits also dropped in 2020 but rebounded to prepandemic weekly levels (2019: mean [range], 634 [56-1703] visits) by the end of 2020 (mean [range], 533 [39-1800] visits).

**Conclusions and Relevance:**

This cross-sectional study suggests that families’ perceptions of the need for emergent MH care were reduced during the pandemic by less than their perception of the need for emergency care overall. Emergency departments should be equipped to provide critical care specifically for adolescents facing MH emergencies.

## Key Points

**Question:**

How did the behavior of adolescents seeking mental health (MH) care in the emergency department (ED) change during the COVID-19 pandemic?

**Findings:**

In this cross-sectional study of ED visits from 2019 through 2021, ED visits and MH-related ED visits dropped following the declaration of the pandemic in March 2020 relative to the same weeks in 2019. While the total number of adolescent ED visits only returned to 2019 levels in the middle of 2021, adolescent MH-related ED visits returned to 2019 levels beginning in late 2020.

**Meaning:**

The rebound of MH-related ED visits to 2019 levels was faster than the rebound of overall ED visits for the adolescent population, which suggests that careful attention should be paid to this population, as there appeared to be less flexibility in the demand for MH care in this age group following the COVID-19 public health emergency.

## Teaser

This cross-sectional study aimed to determine how adolescents, compared with other age groups, sought help in emergency departments in general and for mental health conditions during the COVID-19 pandemic.

## Introduction

The COVID-19 pandemic brought about substantial changes to the global mental health (MH) landscape. Recent literature has reported an increase in adverse mental and behavioral health conditions, including depression, anxiety, substance use, and suicidal ideation during the early months of the pandemic and into 2021 when compared with prior years.^1^ These changes in MH status were widespread, with a 25% increase in the global prevalence of adult anxiety and depression during the first year of the pandemic and considerable increases in rates of major depression, generalized anxiety disorder, acute stress/posttraumatic stress, and suicidal ideation during the first few weeks of the pandemic when compared with years prior.^2^

Emerging literature on adolescent MH during the COVID-19 pandemic points largely toward adverse outcomes for this population as well. Adolescent MH was already worsening in the decade prior to the pandemic; from 2009 to 2019 a 40% increase in persistent feelings of sadness or hopelessness was reported among US high school students.^3^ Shortly after the onset of the pandemic, more than 25% of high school students reported worsened emotional and cognitive health.^4^ Healthy adolescents in some states were found to experience large increases in self-reported anxiety and depression with the onset of the pandemic.^5^ In contrast, a study on a majority Hispanic/Latinx youth population found that MH symptoms were significantly reduced or unchanged after the pandemic began.^6^

Emergency department (ED) visits have previously been used as an important indicator for changes in the MH landscape.^7^ This cross-sectional study examines patterns in MH-related ED visits among adolescents compared with other age groups during the COVID-19 pandemic. There is some existing literature on adolescents presenting to the ED for MH conditions in the early months of the pandemic. For instance, a recent study found that ED visits for suicidality among children and adolescents did not increase following onset of the pandemic.^8^ Another study by the Centers for Disease Control and Prevention (CDC) found that while the absolute number of MH-related ED visits by children decreased, the proportion of MH-related visits increased by 24% and 31% from March 2020 to October 2020 for children aged 5 to 11 years and 12 to 17 years, respectively, compared with the same period in 2019.^9^ Given that MH-related ED visits serve as a proxy for severe MH conditions with unmet needs, any sustained increase in the prevalence of adolescent MH ED visits may indicate MH resource scarcity or that resource allocation is not keeping up with demand.

While these findings offer preliminary insight into pandemic-related changes to pediatric and adolescent MH, less is known about the extent to which adolescents sought ED and MH-related ED care in 2020 and 2021. Recent research suggests that the number of ED visits and MH-related ED visits may have been stable for the US adult population following the pandemic declaration, but both stayed at lower levels than during comparable weeks in the prepandemic period.^10^ Motivated by these patterns, the goal of this study was to quantify changes in the absolute number and proportion of total and MH-related ED visits by adolescents over the course of the pandemic (2019-2021) and compare these patterns to other age groups to assess pandemic-associated changes in the prevalence and severity of adolescent MH conditions.

## Methods

### Data Source

The National Syndromic Surveillance Program (NSSP) mental health data set was provided by the CDC and is based on ED data available as a result of close partnerships with state and local health departments. The data set consists of a panel of weekly data for the 10 US Department of Health and Human Services (HHS) regions. The regions have the following city names as shorthand: Boston, New York, Philadelphia, Atlanta, Chicago, Dallas, Kansas City, Denver, San Francisco, and Seattle. A complete list of states represented by region is available in the eAppendix in Supplement 1. The ED surveillance data are a convenience sample and are not nationally representative. Data were obtained at the weekly level by HHS region for all age cohorts and include ED visit data from 71% of US facilities, where some states have a higher proportion of facilities sending data than others. To limit the affect on data quality, the data set was restricted to facilities reporting more complete data, with an average weekly discharge diagnosis informativeness of 70% or more throughout the analytic period.

This study did not involve human participants and was therefore deemed exempt from review by the University of California, San Francisco Institutional Review Board. This article follows the Strengthening the Reporting of Observational Studies in Epidemiology (STROBE) reporting guidelines for observational studies.

### Definition of Mental Health ED Visits

Mental health ED visits were captured from the NSSP data set using keyword syndromes from the CDC Mental Health, version 1, query, which identifies ED visits where there are acute MH crises (ie, the sole or primary reason for the visit is only related to MH), as well as visits where MH conditions are present (defined as coded in the discharge diagnosis or mentioned in the chief complaint text) but may not be the sole reason for the visit. The complete list of chief complaints and diagnosis codes used to classify visits (outpatient and admitted) into the MH syndrome can be found in the eTable in Supplement 1.

### Statistical Analysis

We investigated patterns of ED-seeking behavior, stratified by age cohort, from 2019 to 2021. The analysis includes the evolution of 3 visit measures: (1) number of total ED visits, (2) number of MH-related ED visits, and (3) the proportion of all ED visits that were MH related. Specifically, we calculated summary statistics for these outcome variables by year and graphically evaluated weekly trends in outcomes by year from January 2019 through December 2021. Analyses were conducted using Stata, version 14.2 (StataCorp). Data analysis was performed in April and May 2023.

## Results

### Age Cohort Trends

**Figure 1.**
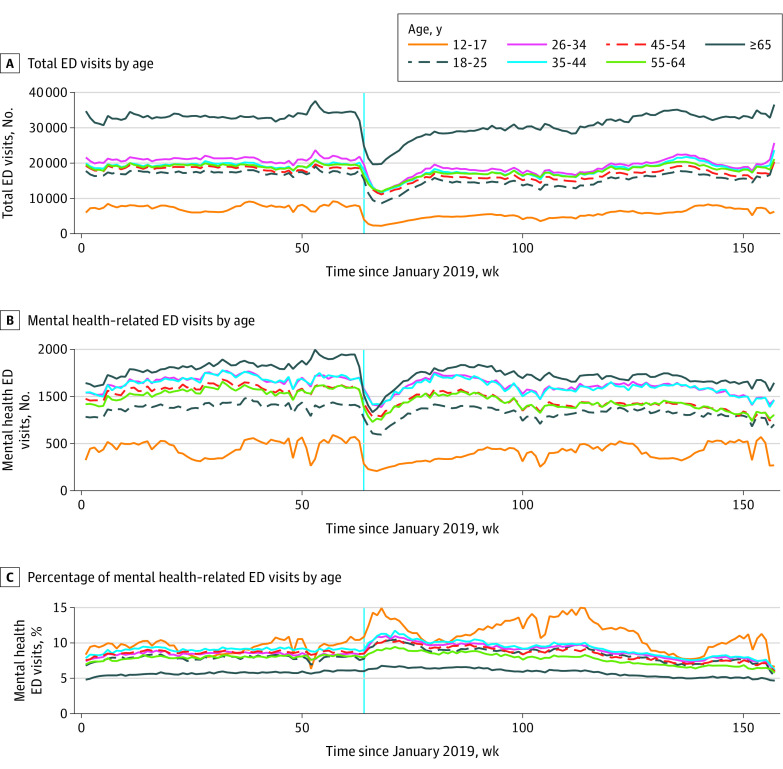
Overall Weekly Trends in Total ED Visits, Mental Health–Related ED Visits, and Percentage of Mental Health–Related ED Visits by Age Cohort Data were obtained from the National Syndromic Surveillance Program data set. The vertical blue line represents the week in March 2020 when the COVID-19 pandemic was declared. ED indicates emergency department.

Figure 1 depicts overall trends in ED visits by age category across the 3 different visit measures: (1) total ED visits, (2) MH-related ED visits, and (3) the proportion of ED visits that were MH-related. Adolescent patients (ages 12-17 years) had the lowest absolute number of total and MH-related ED visits, as shown in Figure 1A and B, respectively. In contrast, the oldest cohort (≥65 years) had the largest number of total and MH-related ED visits.

Figure 1A shows that total ED visits for all age cohorts were fairly stable up until March 11, 2020, the week during which the pandemic was declared. After the pandemic was declared, ED visits for all cohorts dipped but began slowly increasing again throughout the remainder of 2020 and into 2021. Figure 1B shows that MH-related visits followed the same trend for all age cohorts before the pandemic was declared, then dropped steeply for all age cohorts with the onset of the pandemic. Figure 1C reveals that the oldest cohort had the lowest percentage of MH-related visits as a proportion of all ED visits throughout the study period. In contrast, the adolescent category had the highest proportion of MH-related ED visits during most weeks, even before the pandemic was declared. In addition, the adolescent group showed the steepest average increase in the proportion of ED visits that were MH related once the pandemic was declared, from just more than 10% (741 MH-related visits out of 7728 total visits) in the week leading up to the onset of the pandemic in mid-March 2020, to a peak of nearly 15% (319 MH-related visits out of 2252 total visits) 6 weeks after the pandemic declaration (despite the absolute decrease in both categories). This percentage remained elevated throughout subsequent weeks in 2020 and into 2021 relative to the other age cohorts.

### Adolescent Summary Statistics

The Table summarizes the key outcomes of interest from Figure 1 (total ED visits, MH-related ED visits, and proportion of ED visits that were MH related) for the adolescent cohort. Summary statistics for each of the 3 years studied (2019, 2020, and 2021), as well as summary statistics for the entire study period, are included. In 2019, the average number of MH-related ED visits for adolescents was 634 visits, with a minimum of 56 visits and a maximum of 1703 visits per week per HHS region. On average, there were 7358 total ED visits with a minimum of 715 visits and a maximum of 25 908 visits for the adolescent cohort. The percentage of MH-related ED visits was 9.3% in 2019, with a minimum of 4.4% and a maximum of 17.4%.

**Table.** Total Number of ED Visits, MH-Related ED Visits, and Percentage of MH-Related ED Visits by Week and HHS Region Among Adolescent Patients^a^

Year	No.	Mean (SD) [range]
**2019**		
MH-related visits	520	634 (436) [56-1703]
ED visits	520	7358 (5697) [715-25 908]
% MH-related visits	520	9.3 (2.4) [4.4-17.4]
**2020**		
MH-related visits	530	533 (390) [39-1800]
Total ED visits	530	5050 (4426) [445-24 757]
% MH-related visits	530	11.6 (3.2) [5.5-22.8]
**2021**		
MH-related visits	520	617 (4250) [43-1780]
ED visits	520	6210 (4906) [623-25 777]
% MH-related visits	520	10.8 (3.5) [4.9-22.5]
**Total**		
MH-related visits	1570	595 (419) [39-1800]
ED visits	1570	6198 (5118) [445-25 908]
% MH-related visits	1570	10.6 (3.2) [4.4-22.8]

Abbreviations: ED, emergency department; HHS, US Department of Health and Human Services; MH, mental health.

^a^ The unit of observation is specific week in 1 of the 10 HHS regions. There were 520 data points in 2019, 530 in 2020, and 520 in 2021, resulting in 1570 total observations. Data were obtained from the National Syndromic Surveillance Program data set.

In 2020, the average MH-related and total ED visits for the adolescent cohort decreased relative to this same group in 2019 (average MH-related ED visits were 533 in 2020 compared with 634 in 2019, and average total ED visits were 5050 in 2020 compared with 7358 in 2019). However, in 2021 the average number of MH-related and total ED visits rebounded to 617 visits and 6210 visits, respectively. Overall, the percentage of MH-related visits was lowest in 2019 at 9.3% and increased to 11.6% in 2020 before dropping back to 10.8% in 2021.

### Weekly Trends in the Adolescent Cohort

**Figure 2.**
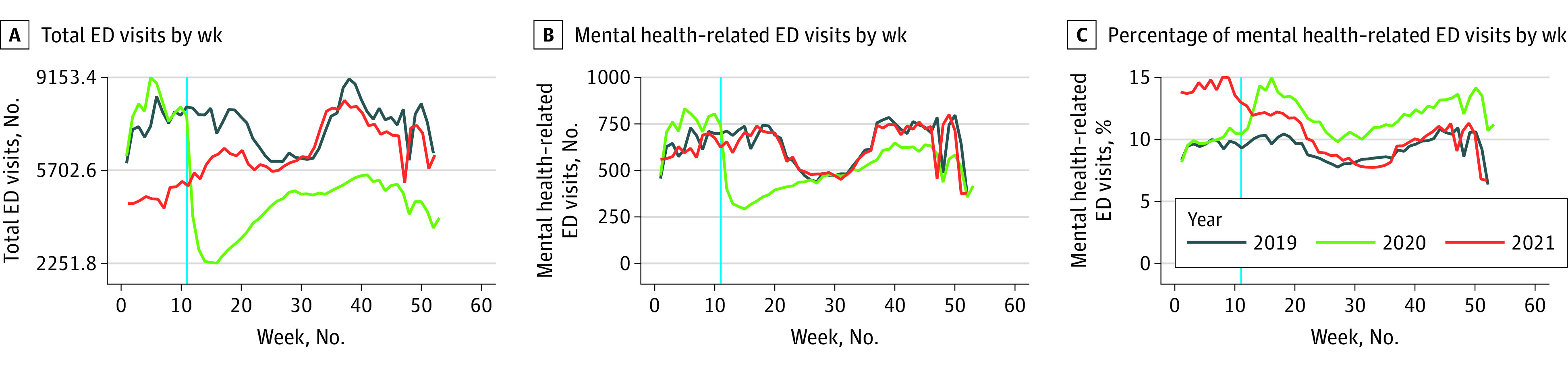
Weekly Trends in Total ED Visits, Mental Health–Related ED Visits, and Percentage of Mental Health–Related ED Visits Averaged Over the 10 HHS Regions for the Adolescent Cohort, 2019-2021 Data were obtained from the National Syndromic Surveillance Program data set. The vertical blue line indicates the week in March 2020 when the COVID-19 pandemic was declared. ED indicates emergency department; HHS, US Department of Health and Human Services.

Figure 2 depicts the weekly evolution of the 3 visit measures for adolescent patients averaged across the 10 HHS regions. Figure 2A depicts total adolescent ED visits by week. Similar trends were observed in 2019 and 2020 in the weeks preceding the second week of March, but the total number of ED visits was higher during the first weeks of 2020 than in the corresponding weeks in 2019. Following the declaration of the COVID-19 pandemic in 2020, the total number of adolescent ED visits plummeted compared with the corresponding weeks in 2019. While the total number of visits increased later in the summer of 2020, it remained much lower than in 2019 for similar weeks. Total adolescent ED visits in 2021 started at a much lower level than in 2020 and 2019 but returned to 2019 levels later that summer.

Trends in the number of adolescent MH-related ED visits are displayed in Figure 2B. The number of adolescent MH-related visits followed a similar pattern in 2019 and 2020 until mid-March, but the number of MH-related visits was higher during this first part of 2020 than during the same period in 2019. Once the pandemic was declared in March 2020, the number of adolescent MH-related ED visits dropped compared with the same weeks in 2019. While the weekly number of MH-related ED visits increased later in the summer of 2020, it did not return to 2019 levels until late 2020. In 2021, adolescent MH-related ED visits tracked closely with 2019 levels during the corresponding weeks.

Figure 2C shows the percentage of all ED visits that were MH-related visits. The 2019 and 2020 weekly trends were nearly parallel up until the week the pandemic was declared in 2020. The percentage of MH-related ED visits then increased in 2020 compared with the same weeks in 2019. The percentage of MH-related ED visits remained high at the beginning of 2021, starting at a much larger percentage than in 2020 and 2019, then slowly dropping in the first half of 2021. In the second half of 2021, percentages returned to and remained at 2019 levels during similar weeks.

## Discussion

This study examining total and MH-related ED visits by adolescents from 2019 to 2021 in the US showed a drop in total ED and MH-related ED visits in 2020 after the onset of the COVID-19 pandemic. While total ED visits only returned to prepandemic weekly levels in the middle of 2021, MH-related visits had rebounded to 2019 weekly levels by late 2020. While visit data varied by region, the overall yearly average proportion of MH-related adolescent ED visits increased from 9.3% in 2019 to 11.6% in 2020 and then down to 10.8% in 2021.

These findings are consistent with other studies that found that the absolute number of pediatric MH ED visits declined during the pandemic when compared with 2019.^9,11^ Another study on adolescent suicide attempts found that while the absolute number of weekly ED visits for suicide attempts dropped just after the pandemic declaration in March and April 2020 compared with the corresponding weeks in 2019, visits subsequently increased by 26.2% in summer 2020 and 50.6% in winter 2021 for girls aged 12 to 17 years compared with 2019 levels.^12^ The present findings extend the study period of these older studies and include a broader group of MH diagnoses, showing that MH-related ED visits returned to prepandemic levels more quickly than total ED visits for adolescents than any other age category.

These results suggest that careful attention should be paid to the MH of this vulnerable group of patients, given that the decrease in total adolescent ED visits during the pandemic was much greater than the decrease in adolescent MH-related ED visits and MH-related visits soon returned to prepandemic weekly patterns. This indicates that adolescent demand for MH care may be less responsive than other types of care, especially during a public health emergency. Given that the observed decrease in ED visits after the declaration of the public health emergency was likely indicative of patients or families delaying care or using alternative forms of care such as telehealth, the smaller decrease and faster rebound in MH ED visits may be due to fewer alternative options for MH care than for other conditions, along with an increased demand for care in the adolescent population, resulting in an greater reliance on the ED during this time period.

These findings may assist policymakers and MH professionals in deciding how to allocate MH resources and funding to procure the greatest benefits. Introducing new federal programs such as the Bipartisan Safer Communities Act of 2022, which provided a large investment in MH funding for children and families across the country,^13^ as well as state or local policy interventions such as California Senate Bill 803, which expanded peer support specialists to Medi-Cal services,^14^ may improve MH care options and reduce the prevalence of MH-related emergencies for adolescents and other vulnerable populations. Sustained, careful implementation of these or other targeted interventions aimed at improving MH resource availability are critical to reducing MH emergencies, particularly in times of crisis.

### Limitations

There are important limitations to this study. First, the data on visits to the ED are provided by week-region. There is no detailed information on what proportion of patients at these visits received care or the diagnosis type for different MH conditions. Second, the classification of near real-time feeds of ED encounters involves text and diagnostic code processing to standardize unstructured fields. Algorithms look for key terms and diagnostic codes while negating other terms to create syndrome categories automatically as new data are processed and may not only include principal diagnoses. Misclassification of encounters during the syndrome classification process is possible due to difficulties identifying or reporting MH-related ED visits. Third, data used in this study includes only visit-level and not patient-level information. It is unknown if these encounters result from incident or recurrent ED visits. We also may underestimate the effect on children if the ED may be the only recourse available for some families. As such, looking solely at MH-related ED visits may not accurately evaluate the burden of disease in children. Fourth, the data do not allow us to distinguish between specific mechanisms driving the MH-related ED visit patterns found in these analyses. For example, we cannot determine whether the increase in proportion of MH-related ED visits was a result of a change in being more willing to seek care vs asymmetric changes in access to care between MH and other health care professionals. Finally, the NSSP ED surveillance data are not a nationally representative sample. Data include ED visits from 71% of US facilities, and facility participation varies within and across states, as well as over time. For instance, fewer than 50% of EDs in California, Hawaii, Iowa, Minnesota, and Oklahoma currently participate, and discharge diagnosis data for Missouri is incomplete. To limit the influence on data quality, the data set was restricted to facilities with an average weekly discharge diagnosis informativeness of 70% or more throughout the analytic period. Future work could access microlevel data to investigate further beyond the aggregate effects found in this study.

## Conclusions

In this cross-sectional study, we found that total and MH-related ED visits dropped after the COVID-19 pandemic declaration in March 2020. While total ED visits for the adolescent group only returned to weekly prepandemic patterns in the middle of 2021, MH-related visits rebounded to prepandemic levels in late 2020. These patterns suggest that EDs, and the broader US health care system, should consider changes in resource allocation that target the specific needs of adolescents and other vulnerable populations, especially in times of crisis.

## Notes

Supplement 1. **eAppendix.** Supporting Information on US Department of Health and Human Services (HHS) Regions **eTable.** Keyword Syndrome Descriptions With Free-Text Reason for Visit (Chief Concern) Search Terms and Diagnosis Codes Included in Keyword Syndromes for Emergency Department Visits Associated With Mental Health Conditions (MHCs)—National Syndromic Surveillance Program

Supplement 2.
Data Sharing Statement
