# Supplementary material for: Adolescent Total and Mental Health–Related Emergency Department Visits During the COVID-19 Pandemic
Source: JAMA Netw Open. 2023 Oct 5;6(10):e2336463. doi: 10.1001/jamanetworkopen.2023.36463 (PMC10556969; doi:10.1001/jamanetworkopen.2023.36463)
Supplement: Supplement 1. — eAppendix. Supporting Information on US Department of Health and Human Services (HHS) Regions eTable. Keyword Syndrome Descriptions With Free-Text Reason for Visit (Chief Concern) Search Terms and Diagnosis Codes Included in Keyword Syndromes for Emergency Department Visits Associated With Mental Health Conditions (MHCs)—National Syndromic Surveillance Program [file jamanetwopen-e2336463-s001.pdf]

Supplemental Online Content

Villas-Boas SB, Kaplan S, White JS, Hsia RY. Adolescent total and mental health–related emergency department visits during the COVID-19 pandemic. *JAMA Netw Open*. 2023;6(10):e2336463. doi:10.1001/jamanetworkopen.2023.36463

eAppendix. Supporting Information on US Department of Health and Human Services (HHS) Regions
eTable. Keyword Syndrome Descriptions With Free-Text Reason for Visit (Chief Concern) Search Terms and Diagnosis Codes Included in Keyword Syndromes for Emergency Department Visits Associated With Mental Health Conditions (MHCs)—National Syndromic Surveillance Program

© 2023 Villas-Boas SB et al. *JAMA Netw Open.*

eAppendix. Supporting Information on US Department of Health and Human Services (HHS) Regions

The 10 HHS regions are as follows:
Region 1 – Boston: Connecticut, Maine, Massachusetts, New Hampshire, Rhode Island, and Vermont
Region 2 - New York: New Jersey, New York, Puerto Rico, and the Virgin Islands
Region 3 – Philadelphia: Delaware, District of Columbia, Maryland, Pennsylvania, Virginia, and West Virginia
Region 4 – Atlanta: Alabama, Florida, Georgia, Kentucky, Mississippi, North Carolina, South Carolina, and Tennessee
Region 5 – Chicago: Illinois, Indiana, Michigan, Minnesota, Ohio, and Wisconsin
Region 6 – Dallas: Arkansas, Louisiana, New Mexico, Oklahoma, and Texas
Region 7 - Kansas City: Iowa, Kansas, Missouri, and Nebraska
Region 8 – Denver: Colorado, Montana, North Dakota, South Dakota, Utah, and Wyoming
Region 9 - San Francisco: Arizona, California, Hawaii, Nevada, American Samoa, Commonwealth of the Northern Mariana Islands, Federated States of Micronesia, Guam, Marshall Islands, and Republic of Palau
Region 10 – Seattle: Alaska, Idaho, Oregon, and Washington

© 2023 Villas-Boas SB et al. *JAMA Netw Open.*

eTable. Keyword Syndrome Descriptions With Free-Text Reason for Visit (Chief Concern) Search Terms and Diagnosis Codes Included in Keyword Syndromes for Emergency Department Visits Associated With Mental Health Conditions (MHCs)—National Syndromic Surveillance Program

Definition	Chief Complaint Search Terms	Diagnosis Codes
**CDC Overall Mental Health Conditions v1**	Schizophrenia (include misspellings)<br>Schizotypal (include misspellings)<br>Schizoaffective (include misspellings)<br>Grief (include misspellings)<br>Grieve (include misspellings)<br>Bereave (include misspellings)<br>Psych<br>Mental health<br>Mental evaluation<br>Mental problem<br>Mentally<br>Mental illness<br>Reactive attachment<br>Auditory hallucination<br>Visual hallucination<br>Hearing voice<br>Evaluation hallucination<br>History of hallucination<br>Hallucinations patient<br>Hallucinations unspecified<br>Seeing thing<br>Delusion (include misspellings)<br>Paranoia (include misspellings)<br>Trichotillomania<br>Dissociative disorder<br>Dissociative conversion<br>Dissociative identity<br>Dissociative and conversion<br>Dissociative tendency<br>Dissociative episode<br>Dissociative conversion<br>Dissociative behavior<br>Dissociative amnesia<br>Dissociative attentive<br>Experiencing dissociation<br>Dissociative (when used with depress, depression, psych, dysthymic, anxiety, anxious, traumatic stress, PTSD, adjustment disorder, mental, attention deficit, bipolar, schizophrenia, schizoaffective, schizotypal – and associated misspellings of all terms)	295.00; 295.01; 295.02; 295.03; 295.04; 295.10; 295.11; 295.12; 295.13; 295.14; 295.20; 295.21; 295.22; 295.23; 295.24; 295.30; 295.31; 295.32; 295.33; 295.34; 295.40; 295.41; 295.42; 295.43; 295.44; 295.50; 295.51; 295.52; 295.53; 295.54; 295.60; 295.61; 295.62; 295.63; 295.64; 295.70; 295.71; 295.72; 295.73; 295.74; 295.80; 295.81; 295.82; 295.83; 295.84; 295.90; 295.91; 295.92; 295.93; 295.94296.90; 296.99; 297.0; 297.1; 297.2; 297.3; 297.8; 297.9; 298.0; 298.1; 298.2; 298.3; 298.4; 298.8; 298.9; 300.10; 300.11; 300.12; 300.13; 399.14; 300.15; 300.81; 300.82; 300.89; 300.7; 307.80; 307.89; 300.9 648.40; 648.41, 648.42, 648.43; 648.44; V62.82<br><br>F20; F21; F22; F23; F25; F28; F29; F39; F44; F45; F48; F53.1; F54; F63.3; F93.8; F93.9; F94; F98.9; F99; Z63.4<br><br>413307004; 74732009; 231538003; 268664001; 48500005; 191667009; 41526007; 18193002; 224965009; 74506000; 58214004; 268617001; 64905009;191526005; 68890003

© 2023 Villas-Boas SB et al. *JAMA Netw Open.*

**CDC Depressive Disorders v1**	Depression<br>Dysthymic<br>Dysthymic<br>Mood disorder<br>Mood disturbance	296.20; 296.21; 296.22; 296.23; 296.24; 296.25; 296.30; 296.31; 296.32; 296.33; 296.34; 296.35; 300.4; 309.0; 309.1; 296.90; 296.99; 309.28<br><br>F32 (except F32.5); F33 (except F33.42); F34.1; F34.9; F43.21; F43.23; O90.6<br><br>35489007; 310497006; 370143000; 712823008; 57194009
**CDC Attention-Deficit/Hyperactivity Disorders v1**	Attention deficit<br>Hyperactivity<br>Attention problems	314.00, 314.01; 314.9; 314.2<br><br>F90 (include all in this series)<br><br>406506008<br>35253001
**CDC Trauma and Stressor-related Disorders v1**	Traumatic stress<br>PTSD (including misspellings)<br>Adjustment disorder<br>Reaction to acute stress<br>Acute stress reaction<br>Acute stress crisis<br>Acute stress eval<br>Eval acute stress<br>Acute stress behavior (including misspellings for behavior)<br>Behavior acute stress<br>Behavior acute stress<br>Reaction to severe stress<br>Severe stress reaction<br>Severe stress crisis	308.0; 308.1; 308.2; 308.3; 308.4; 308.9; 309.81; 309.89; 309.9; 309.0; 309.1; 309.24; 309.28;309.29; 309.3; 309.4<br><br>F43<br><br>67195008; 47505003; 17226007; 57194009; 271952001
**CDC Disruptive Behavioral and Impulse-Control v1**	Conduct disorder<br>Oppositional defiant (with spelling mistakes for "defiant")<br>Antisocial personality<br>Disturbance of conduct<br>Impulse control disorder<br>Explosive disorder	309.3; 309.4; 312.00; 312.01; 312.02; 312.03; 312.10; 312.11; 312.12; 312.13; 312.20, 312.21; 312.22; 312.23; 312.4; 312.81; 312.82; 312.89; "312.9; 312.30; 312.31; 312.39; 313.81; 314.2; 301.7<br><br>F43.24; F43.25; F91.0; F91.1; F91.2; F91.3; F91.8; F91.9; F63.81; F63.1; F63.2; F63.9; F63.0; F63.89; F60.2<br><br>54319003; 18941000; 430909002; 26665006

© 2023 Villas-Boas SB et al. *JAMA Netw Open.*

**CDC Bipolar Disorders v1**	Bipolar<br>Manic<br>Cyclothymic<br>Mania<br>Hypomania<br>Hypermania	296.00; 296.01; 296.02; 296.03; 296.04; 296.05; 296.40; 296.41; 296.42; 296.43; 296.44; 296.45; 296.50; 296.51; 296.52; 296.53; 296.54; 296.55; 296.60; 296.61; 296.62; 296.63; 296.64; 296.65; 296.7<br>296.80; 296.81; 296.82; 296.89<br><br>F30.1; F30.2; F30.3; F30.8; F30.9; F31.0; F31.1; F31.2; F31.3; F31.4; F31.5; F31.6; F31.7 (only include: F31.70, F31.71, F31.73, F31.75, F31.77); F31.8; F31.9; F34.0<br><br>191627008; 371596008; 83225003; 231494001; 284513006; 268619003
**CDC Eating Disorders v1**	Pica<br>Bulimia (including misspellings)<br>Binge eating (including misspellings of binge)<br>Binge-eating<br>Eating disorder<br>Rumination disorder<br>Binging or purging<br>Binging and purging<br>Weight loss or anorexia<br>Anorexia nervosa (including misspellings of anorexia)<br>Anorexic (including misspellings of anorexic)<br>Being treated for anorexia (including misspellings of anorexia)<br>Treatment for (including misspellings of anorexia)<br>Weight loss (including misspellings of weight; include when used with anorexia)<br>Loss of weight (including misspellings of weight; include when used with anorexia)<br>Weight loss (including misspellings of weight; include when used with anorexia)<br>Loss of weight (including misspellings of weight; include when used with anorexia)<br>Eating disorder<br>Anorexia history (including misspellings of anorexia)<br>History of anorexia (including misspellings of anorexia)<br>Mental anorexia (including misspellings of anorexia)<br>Mental (when used with anorexia)<br>Psych (when used with anorexia)	307.1; 307.50; 307.51; 307.52; 307.53; 307.54; 307.59<br><br>F50.00; F50.01; F50.02; F50.2; F50.8; F50.82; F50.89; F50.9; F98.21; F98.29; F98.3<br><br>72366004; 56882008; 7800400; 32721004

© 2023 Villas-Boas SB et al. *JAMA Netw Open.*

**CDC Tic Disorders v1**	Neurological tic<br>Tic disorder<br>Behavioral tic<br>Facial tic<br>Face tic	307.20; 307.21; 307.22; 307.23<br><br>F95; G25.69<br><br>568005; 5158005
**CDC Obsessive-Compulsive Disorders v1**	Obsessive compulsive<br>Compulsive disorder	300.3<br><br>F42<br><br>191736004

© 2023 Villas-Boas SB et al. *JAMA Netw Open.*
