# Supplementary material for: Adolescent Total and Mental Health–Related Emergency Department Visits During the COVID-19 Pandemic
Source: JAMA Netw Open. 2023 Oct 5;6(10):e2336463. doi: 10.1001/jamanetworkopen.2023.36463 (PMC10556969; doi:10.1001/jamanetworkopen.2023.36463)
Supplement: Supplement 2. — Data Sharing Statement [file jamanetwopen-e2336463-s002.pdf]

Data Sharing Statement

Villas-Boas. Adolescent Total and Mental Health–Related Emergency Department Visits During the COVID-19 Pandemic. *JAMA Netw Open*. Published October 05, 2023. doi:10.1001/jamanetworkopen.2023.36463

Data
Data available: No
